# The Route of Sucrose Utilization by *Streptococcus mutans* Affects Intracellular Polysaccharide Metabolism

**DOI:** 10.3389/fmicb.2021.636684

**Published:** 2021-02-02

**Authors:** Bárbara Emanoele Costa Oliveira, Antônio Pedro Ricomini Filho, Robert A. Burne, Lin Zeng

**Affiliations:** ^1^Department of Oral Biology, College of Dentistry, University of Florida, Gainesville, FL, United States; ^2^Department of Biosciences, Piracicaba Dental School, University of Campinas, Piracicaba, Brazil

**Keywords:** carbohydrate starvation, Streptococcus mutans, IPS, dental caries, gene expression, sucrose metabolism

## Abstract

*Streptococcus mutans* converts extracellular sucrose (Suc) into exopolysaccharides (EPS) by glucosyl-transferase and fructosyl-transferase enzymes and internalizes Suc for fermentation through the phosphotransferase system (PTS). Here, we examined how altering the routes for sucrose utilization impacts intracellular polysaccharide [IPS; glycogen, (*glg*)] metabolism during carbohydrate starvation. Strain UA159 (WT), a mutant lacking all exo-enzymes for sucrose utilization (MMZ952), and a CcpA-deficient mutant (*∆ccpA*) were cultured with sucrose or a combination of glucose and fructose, followed by carbohydrate starvation. At baseline (0h), and after 4 and 24h of starvation, cells were evaluated for mRNA levels of the *glg* operon, IPS storage, glucose-1-phosphate (G1P) concentrations, viability, and PTS activities. A pH drop assay was performed in the absence of carbohydrates at the baseline to measure acid production. We observed *glg* operon activation in response to starvation (*p*<0.05) in all strains, however, such activation was significantly delayed and reduced in magnitude when EPS synthesis was involved (*p*<0.05). Enhanced acidification and greater G1P concentrations were observed in the sucrose-treated group, but mostly in strains capable of producing EPS (*p*<0.05). Importantly, only the WT exposed to sucrose was able to synthesize IPS during starvation. Contrary to CcpA-proficient strains, IPS was progressively degraded during starvation in *∆ccpA*, which also showed increased *glg* operon expression and greater PTS activities at baseline. Therefore, sucrose metabolism by secreted enzymes affects the capacity of *S. mutans* in synthesizing IPS and converting it into organic acids, without necessarily inducing greater expression of the *glg* operon.

## Introduction

Cariogenic biofilms are formed under a dynamic condition of exposure to high concentrations of carbohydrates (feast), followed by periods of nutrient limitation (famine; Carlsson, 1983). Under feast conditions, the major etiological agent of dental caries, *Streptococcus mutans*, is able to produce organic acids and also to convert the excess carbohydrates into intracellular polysaccharides (IPS; Huis In’t Veld and Backer Dirks, 1978; Wilson et al., 2010), a glycogen (*glg*)-like molecule composed primarily of α-1,4-linked glucose polymers. Past research suggests that IPS could play an important role as a storage compound (Preiss, 2006; Busuioc et al., 2009), as *S. mutans* enzymes subsequently break down IPS and release glucose once extracellular carbohydrate sources have been depleted. Thus, IPS is expected to promote bacterial survival, especially during carbohydrate starvation periods (Spatafora et al., 1995; Busuioc et al., 2009; Demonte et al., 2017). Importantly, IPS has been shown to contribute to cariogenicity in animal models, likely by extending the extent and duration of acid production (Gibbons and Socransky, 1962; Harris et al., 1992; Spatafora et al., 1995), leading to further tooth demineralization even when dietary carbohydrates are absent.

**Figure 1:**
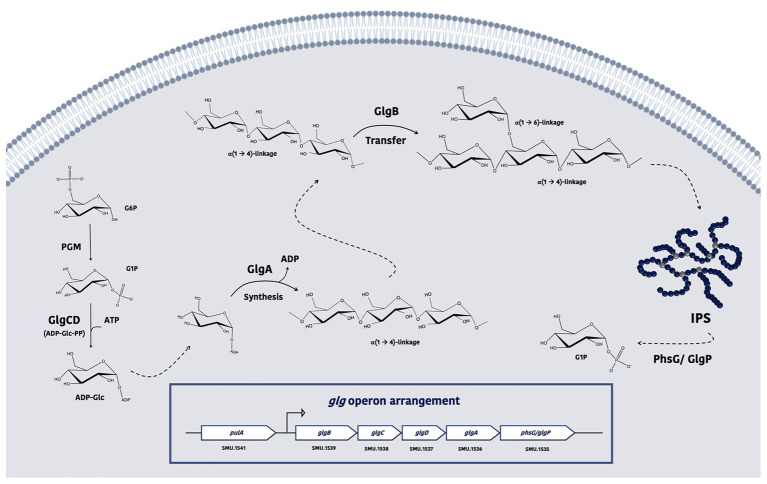
A model depicting the proposed functions of enzymes required for intracellular polysaccharide (IPS) metabolism in *S. mutans*. Glucose-6-phosphate (G6P) is converted by phosphoglucomutase (PGM) into glucose-1-phosphate (G1P), which can then be converted into ADP-Glc *via* the action of ADP-Glc pyrophosphorylase (PP), which is comprised of GlgC and GlgD. As the donor of the subunits for IPS, ADP-Glc is taken by glycogen (*glg*) synthase GlgA and used to elongate a nascent strand of glucose polymer, forming α-1,4-linkages. This product is further acted upon by the branching enzyme GlgB to form branches by altering its α-glucosidic linkages (into α-1,6 bonds). When triggered by the energy needs of the bacterium, the activity of *glg* phosphorylase GlgP/PhsG can hydrolyze IPS and release the glucose as G6P.

According to available genetic evidence, enzymes responsible for IPS synthesis (Figure 1) and degradation are encoded in the *glg* operon (SMU.1535-SMU.1539; Harris et al., 1992; Spatafora et al., 1995), which includes *glgA* (glycogen synthase; Busuioc et al., 2009), *glgB* (branching enzyme; Kim et al., 2008), *glg*C&*D* (two subunits of the ADP-Glc-pyrophosphorylase, ADP-Glc-PP; Diez et al., 2013; Demonte et al., 2017), and *glgP* (or *phsG*, glycogen phosphorylase). The primary regulatory point for IPS biosynthesis is the creation of ADP-glucose, a reaction catalyzed by ADP-Glc-PP and allosterically regulated by glycolytic intermediates, including fructose-6-phosphate (F6P) or fructose-1,6-bisphosphate (F-1,6-bP) for activation, and AMP, ADP, or Pi for inhibition (Ballicora et al., 2003); corresponding to conditions of high and low energy supply, respectively. Perhaps paradoxically, accumulation of IPS in *S. mutans* has been shown to occur in the late exponential or stationary phase of growth of batch cultures, or under nutrient deprivation conditions (Harris et al., 1992). Recent studies have demonstrated that the transcript levels of the *glg* operon are increased under glucose-limiting as opposed to glucose-excess conditions (Moye et al., 2014), yet the presence of sucrose (Suc) or fructose tends to reduce its expression when compared to glucose (Zeng and Burne, 2016). On the other hand, direct measurements of IPS showed higher amounts of IPS when cariogenic biofilms were exposed to Suc than glucose+fructose control (Oliveira et al., 2017). These findings highlight the fact that the mechanisms regulating *glg* gene expression and IPS metabolism are not well understood in *S. mutans*.

As perhaps the most cariogenic carbohydrate, sucrose can be converted to soluble and insoluble exopolysaccharides (EPS) by glucosyltransferases (GtfBCD, forming homopolymers of glucose called glucans) and fructosyltransferase (Ftf, forming homopolymers of fructose termed fructans) secreted by *S. mutans* (Bowen and Koo, 2011), and can be internalized for fermentation through the sugar: phosphotransferase system (PTS; Slee and Tanzer, 1982; Tao et al., 1993). The glucans promote bacterial attachment and biofilm accumulation, whereas fructans serve primarily as extracellular storage polysaccharides that contribute to the persistence and cariogenicity of *S. mutans* (Staat and Schachtele, 1974; Burne et al., 1996; Khalikova et al., 2005; Klahan et al., 2018). Activities of these exo-enzymes, as well as internalization of sucrose *via* the PTS have been shown to profoundly influence bacterial gene regulation, both through the release of monosaccharides, i.e., glucose and fructose, and PTS-dependent regulation of gene expression (Zeng and Burne, 2013, 2016). Earlier studies on IPS metabolism by *S. mutans* demonstrated an influence of carbohydrate catabolite repression (CCR) and PTS on *glg* gene expression (Spatafora et al., 1999; Zeng et al., 2013). Specifically, the catabolite control protein A (CcpA) is a major CCR regulator required for balancing growth, virulence expression, and bacterial persistence, and a *ccpA* mutant of *S. mutans* had increased acid production from intracellular reservoirs and derepressed expression of the *glg* operon, likely through controlling both energy conservation and production (Abranches et al., 2008; Zeng et al., 2013). Furthermore, a *dlt* (D-alanine-activating enzymes) cluster that is likely involved in the biosynthesis of D-alanyl-lipoteichoic acid affects *glg* expression in response to carbohydrates that are internalized by the PTS (Spatafora et al., 1999). Considering all these observations, we hypothesized that IPS biosynthesis and degradation is regulated by nutrient availability and specific carbohydrate source, and that sucrose could impact IPS metabolism in ways that extend beyond simply serving as a source of hexoses to be incorporated into IPS.

By applying genetic and biochemical analyses to *S. mutans* UA159 and two previously-defined genetic mutants, we examined how altering the routes of sucrose utilization impacts the physiology of *S. mutans* and regulation of *glg* genes under carbohydrate starvation conditions. The results revealed a novel impact of EPS biosynthesis on IPS metabolism that influences bacterial metabolic properties and virulence-related traits.

## Materials and Methods

### Experimental Design

An *in vitro* study was conducted using bacterial batch cultures of (i) *S. mutans* UA159 wild type as control and its otherwise-isogenic mutants, (ii) MMZ952, which lacks all exo-enzymes for sucrose utilization, and (iii) a derivative lacking the global regulator CcpA (*∆ccpA*; Table 1). After growing overnight in Brain Heart Infusion (BHI), cultures were diluted 1:10 into Tryptone-Yeast extract (TY) medium containing (i) 0.5% glucose+0.5% fructose (Glu+Fru) or (ii) 1% Suc at 37°C in an aerobic atmosphere with 5% CO_2_. Upon reaching OD_600_=0.5, cells were collected, washed, and resuspended in fresh TY without carbohydrates. After 4 and 24h of carbohydrate starvation, IPS levels were quantified by use of an iodine-based assay (DiPersio et al., 1974), and viable cells were determined by CFU enumeration. Quantitative real-time PCR (qRT-PCR) was performed to quantify the mRNA levels of the genes involved in IPS metabolism (Table 2 for primers). Cells were visualized by transmission electron microscopy (TEM) to assess IPS content (DiPersio et al., 1974, modified) and glucose-1-phosphate (G1P) concentrations were measured. Additionally, cultures grown to mid-exponential phase (OD_600_=0.5) in TY-based media were used for evaluating the sugar-specific PTS activity, as described elsewhere (LeBlanc et al., 1979). Also, pH values of bacterial cultures were recorded for 60min to assess the ability of the bacteria in lowering the pH at the expense of intracellular energy storage (Ahn et al., 2009; Moye et al., 2014). Experiments were each performed in biological triplicates and results were analyzed at a significance level of 0.05.

**Table 1:** Bacterial strains used in this study.

Strain	Characteristics	Reference
UA159	*Streptococcus mutans* wild-type reference strain	University of Alabama
MMZ952	*gtfA*::Em *gtfBCD ftf fruA*	Zeng and Burne, 2013
∆*ccpA*	*ccpA*::Em	Wen and Burne, 2002; Abranches et al., 2008

**Table 2:** Primers used in this study.

Target	Description/purpose	Primer name	Sequence (5'**→** 3')
SMU.1536	Putative bacterial glycogen synthase GlgA, qRT-PCR.	glgA F	ACGATCTGCATAGAGCACCG
		glgA R	TGGAGTTGGTGATGAGCGTT
SMU.1539	Putative 1,4-alpha-glucan branching enzyme GlgB, qRT-PCR.	glgB F	GTAAAGTGACCAGGCACCCA
		glgB R	GGGCTTCTTTGCCTTTGAGC
SMU.1538	GlgC; putative glucose-1-phosphate adenylyltransferase; ADP-glucose pyrophosphorylase, qRT-PCR.	glgC F	TCAACAAGCATGGTCCGTAA
		glgC R	TCCAATGACCGTATCGTTGA
SMU.1537	Putative glycogen biosynthesis protein GlgD, qRT-PCR.	glgD F	TGGCAGTTGCGCGAAATAAT
		glgD R	AGCGACCTATTTTGCAGTGGA
SMU.1535	Glycogen phosphorylase PhsG, qRT-PCR.	phsG F	TCAATGATACCCATCCAGCA
		phsG R	CTCATCGTTTGCACAGTCGT
*gyrA*	DNA gyrase A subunit, internal control for qRT-PCR.	gyrA F	CCAAGAATCTGCTGTCCG
		gyrA R	TTGCGACTATCTGCTATGTG

### Bacterial Culture Conditions and Starvation Assay

*Streptococcus mutans* strains were maintained on BHI agar plates (Difco, Detroit, MI). For bacterial starvation assays, strains were cultured overnight in BHI, with the appropriate antibiotic added when needed, and sub-cultured into Tryptone-Yeast extract medium supplemented with 0.5% (w/v) each of glucose and fructose or 1% sucrose. Cultures were incubated (37°C, 5% CO_2_), and upon reaching mid-exponential phase (OD_600_=0.5; see Supplementary Figure S1 for growth curves), cells were harvested by centrifugation (4°C, 4,000×*g*, 10min), washed twice in saline solution, resuspended in fresh TY with no added carbohydrates (starvation conditions) and incubated for 24h. At baseline (0h), and after 4 and 24h of carbohydrate starvation, cultures were harvested and samples were either used immediately or stored at −80°C for further analyses.

### Determination of Viable Cells and IPS Levels

At the specified time points, 10ml of cultures were collected and immediately used in the assays. After sonication (FB120, Fisher Scientific; 100% power, 30s), 100μl of the cell suspensions were used for serial dilution and plated onto BHI agar plates for CFU enumeration. For assessing IPS content in the same samples, the remaining suspensions were heated in a water bath at 100°C for 5min to inactivate enzymes and cells were collected by centrifugation (4,000×*g*, 10min; DiPersio et al., 1974; Busuioc et al., 2009). Suspensions were washed twice with ice-cold water and resuspended in 1ml of water in 15-ml conical tubes. After adding 0.3ml of 5.3M KOH, tubes were placed in a boiling water bath for 90min. After cooling, the clear solutions were neutralized by adding 0.3ml of 5.3M HCl followed by 1.0ml of 1.0M potassium phosphate (pH 7.0; Busuioc et al., 2009; Demonte et al., 2017). After mixing, 0.6ml of fresh iodine assay solution (0.2% iodine in 2.0% potassium iodide, Fisher) was added to each tube and the absorbance at 520nm was measured using a spectrophotometer. Purified water was used as blank. A standard curve prepared using a 1mg/ml glycogen (Sigma) stock was used for calculating IPS concentration in samples.

### Quantitative Real-Time PCR

After 0, 4, or 24h of starvation, 10ml of the cultures were centrifuged and treated with 1ml RNAprotect Bacteria Reagent (Qiagen) before storage at −80°C. Total RNA was extracted as described elsewhere (Ahn et al., 2005), and cDNA was synthesized from 0.5μg of RNA using the iScript Select cDNA Synthesis kit (Bio-Rad). The resultant cDNAs were diluted 1:10 with water and used as templates for quantitative PCR (qPCR) for gene expression analysis, using gene-specific primers (Table 2). qPCR was performed using an iCycler iQ real-time PCR detection system (CFX96; Bio-Rad) and iQ SYBR green Supermix (Bio-Rad) according to the protocols provided by the suppliers. Each assay was performed with biological and technical triplicates. For each gene, the mRNA levels were normalized against that of *gyrA* transcript (Vujanac et al., 2015; Zeng and Burne, 2016) using the ∆∆Cq method (Schmittgen and Livak, 2008; Rocha et al., 2015) and presented as relative abundance in comparison to the results of the control group (Glu+Fru) at time 0h.

### Transmission Electron Microscopy

An aliquot of 1ml of culture for each harvest time was collected by centrifugation at 15,000×*g* at room temperature (RT) for 1min and used for TEM analysis. Cells were washed with 0.1M sodium cacodylate (NaCac) buffer and fixed with 3% glutaraldehyde for 60min at RT, followed by an overnight fixation at 4°C. Cells were washed three times with 0.1M NaCac buffer to remove fixative solution, followed by a post-fixation with 1.5% K_4_[Fe(CN)_6_] and 1%OsO_4_ in NaCac buffer (4°C, 60min). Cells were then washed with the same buffer before an equal volume of melted 5% agarose (in PBS) was added to the samples. After cooling (4°C, 2h), pellets were trimmed into thin pieces for graded dehydration with 30–100% ethanol (twice, RT, 10min each). Transition to epoxy resin was done by immersing samples into propylene oxide 100%, followed by epoxy/propylene mixture (1:1) overnight. Samples were then polymerized into 100% epoxy at 60°C for 48h, and then ultrathin sections were prepared (Leica Ultracut UCT ultramicrotome) and mounted on formvar-carbon-coated nickel grids. For IPS visualization, a protocol described by DiPersio et al. (1974) was modified and used. Briefly, the grids were treated with a 1% periodic acid solution (RT, 30min), followed by a sodium chlorite solution treatment (RT, 10min; DiPersio et al., 1974). Grids were washed in purified water, dried and poststained with saturated alcoholic uranyl acetate solution (8% in 50% ETOH) and 0.25% aqueous lead citrate. Grids were visualized using a Hitachi H7600 and JEOL JEM 1400 transmission electron microscopes at 80kV (Sampaio et al., 2019).

### G1P Assay

At each starvation harvest time (0, 4, or 24h), 5ml of cultures were collected, immediately centrifuged (4,000×*g*, 4°C, 10min) and stored at −80°C. For G1P measurement, a G1P Colorimetric Assay Kit (Sigma) was used and samples were prepared as recommended by the supplier. Briefly, cells were resuspended in 200μl of ice-cold G1P Assay Buffer. After incubation (4°C, 10min), samples were collected by centrifugation (15,000×*g*, 10min) and 50μl of the supernates were used for G1P measurements in duplicate. A standard curve generated from 0 (blank) to 10nmol G1P/well. After reaction, measurements were performed in flat-bottom 96-well plates at 450nm, and an area under the curve (AUC) was calculated.

### PTS Assay

Carbohydrate-specific PTS activity was assessed using cultures of *S. mutans* UA159, MMZ952 and *∆ccpA* harvested at mid-exponential phase (OD_600_=0.5). After thawing on ice, cells were washed twice in 0.1M sodium-potassium phosphate buffer (pH 7.2) containing 5mM MgCl_2_ and resuspended in 10% of the initial volume using the same buffer. Cell permeabilization was achieved by vortexting with 0.05 volumes of toluene-acetone solution (1:9; vol/vol), and 50μl of the resultant suspension was used in each reaction. The 1-ml reaction mixture included 0.1M sodium potassium phosphate buffer, 5mM MgCl_2_, 100μM NADH, 10mM NaF, 10mM of Glu+Fru or Suc, 10 units of a lactate dehydrogenase solution (Sigma), and 5mM PEP (0 for the blank; LeBlanc et al., 1979; Moye et al., 2014). The reaction was conducted at 37°C and the rate of PEP-dependent NADH oxidation was evaluated over time by monitoring the optical density (340nm). Protein concentration was assessed using a bicinchoninic acid (BCA) assay (Sigma). Activity was expressed as nmol NADH oxidized (min)^−1^ (mg of protein)^−1^.

### pH Drop Assay

The capacity of cells to lower the pH in the absence of carbohydrates was monitored by performing a pH drop assay (Bender et al., 1986; Ahn et al., 2009). Cultures of *S. mutans* were grown to OD_600_=0.5, washed twice in 1 volume of ice-cold sterile water (4°C, 4,000×*g*, 10min) and resuspended in 5ml of 50mM KCl, 1mM MgCl_2_. The suspensions were titrated with 0.1M KOH to adjust the pH to 7.2. Immediately upon reaching a value of 7.2 (initial pH), the pH was recorded for 60min using a pH meter connected to a computer. Subsequently, the pH values were converted into H^+^ concentration and an AUC was calculated.

### Statistical Analysis

Unless specified otherwise, all experiments were performed using at least three biological repeats. The Kolmogorov-Smirnov test was used for checking the assumptions of normal distribution of errors and homogeneity of variances. Data were analyzed by two-way ANOVA of repeated measurements, considering carbohydrate factor in 2 levels (Glu+Fru or Suc) and biofilm harvest moment in 3 levels (0, 4, and 24h of starvation), followed by Dunnet test (Supplementary Tables S1 and S2). One-way ANOVA followed by Tukey’s test was used for AUC data (G1P and H^+^ concentrations). GraphPad Software (GraphPad Prism, La Jolla, CA, United States) was used and a significance cutoff of 0.05 was selected for all analyses.

## Results

### *glg* Genes Are Activated During Starvation and Expression Patterns Are Influenced by EPS Synthetic Ability

**Figure 2:**
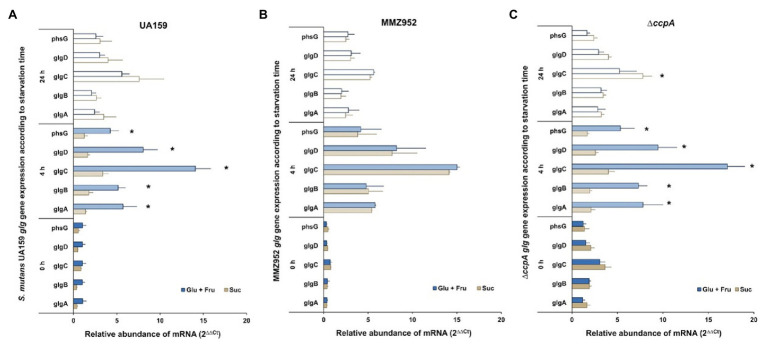
Relative abundance of mRNAs of the *glg* operon expressed by *S. mutans* pre-starvation and post-starvation. The data are arranged according to the carbohydrates used in TY media (Glu+Fru or Suc) and periods of starvation treatment (0, 4, and 24h), and evaluated in the strains of UA159 WT **(A)**, MMZ952 **(B)** and ∆*ccpA* (**C**; Two-way ANOVA; Mean±SD; *n*=3; *p*<0.05). Asterisks indicate statistically significant differences between carbohydrate treatments for each starvation time.

To assess the effects of the routes of sucrose utilization on IPS metabolism, gene expression profiles of the *glg* operon during carbohydrate starvation were obtained by applying qRT-PCR on samples grown on 1% Suc or equivalent amounts (w/v) of equal concentrations (0.5%) of glucose and fructose (Glu+Fru). The analysis was performed on UA159 (WT) and two mutant strains, MMZ952 and *∆ccpA*. For the WT cultured with Glu+Fru, expression of the *glg* operon was greatly enhanced at 4h of starvation, and declined at 24h, ending at levels that were still significantly higher than those at baseline (*p*<0.05; Figure 2A; Supplementary Table S1). This pattern of change was generally true across the entire operon. However, this was not the case for the WT cultured on Suc. Specifically, at baseline and after 4h of starvation, expression of the *glg* operon was significantly lower in WT cells grown with Suc than with Glu+Fru, although not all points of comparison showed statistical significance. Conversely, in comparison to Glu+Fru, despite lower activation of the operon by Suc after 4h of starvation, the upward trend in *glg* expression levels appeared to continue for Suc-grown cells throughout the 24-h starvation period. After 24h of starvation treatment, little difference was noted in *glg* expression levels between Glu+Fru-grown and Suc-grown cells. Thus, expression of the *glg* operon during starvation proceeds by a different trajectory in WT *S. mutans* cultivated with sucrose, potentially due to PTS-dependent effects or related to the activities of a suite of enzymes secreted by UA159 that act upon sucrose (Rölla, 1989; Leme et al., 2006).

In support of the hypothesis that EPS synthesis may impact IPS metabolism, loss of the sucrolytic exo-enzymes (in MMZ952), GtfB, GtfC, GtfD, Ftf, and FruA (Zeng and Burne, 2013), resulted in altered expression of the *glg* operon when grown on sucrose (Figure 2B). Specifically, and unlike for the WT, no difference was observed when the results from Glu+Fru-grown MMZ952 cells were compared with those from the same strain grown on Suc (*p*>0.05), regardless of the duration of exposure to starvation conditions. In other words, the sucrose-dependent phenotype observed in the WT (Figure 2A) requires at least some of the exo-enzymes missing from MMZ952, whereas loss of the exo-enzymes had no impact on *glg* expression in cells grown on Glu+Fru. Interestingly, for the CcpA-deficient mutant at baseline, there was a general increase in the levels of *glg* expression compared to the WT, particularly for Suc-grown cells, and a lack of difference in *glg* expression between the two carbohydrate conditions for the *ccpA* mutant (Figure 2C). After 4h of starvation, a significant increase in *glg* expression was noted for the *ccpA* mutant relative to baseline, but mainly under Glu+Fru conditions, and the difference between carbohydrate conditions resembled that of the WT. For *∆ccpA*, the overall trend in expression of the *glg* operon under Glu+Fru conditions throughout the 24h was similar to that of the WT. However, due to enhanced expression at baseline, the *glg* operon in *∆ccpA* grown on Suc presented a delayed activation, showing significant difference only after 24h of starvation (Supplementary Table S1; *p*<0.05). The enhanced *glg* expression in the *ccpA* mutant at baseline is consistent with prior transcriptomic studies with the same mutant (Abranches et al., 2008; Zeng et al., 2013); however, the delay in *glg* activation after starvation in Suc-grown cells suggests the involvement of additional sucrose-specific mechanisms. Interestingly, for both the genes for the biosynthetic (GlgABCD) and degradative enzymes (PhsG/GlgP) of the glycogen pathway, their transcription regulation proceeded in the same direction for all strains evaluated, likely because they are all in an operonic organization. Consequently, there must be some level of post-transcriptional control of IPS synthesis and degradation, e.g., modification of enzymatic activities *via* allosteric effectors, if accumulation and catabolism of IPS is to be properly coordinated.

### IPS Synthesis and Degradation Are Affected by Carbohydrate Source, Ability to Synthesize EPS, and CcpA

**Figure 3:**
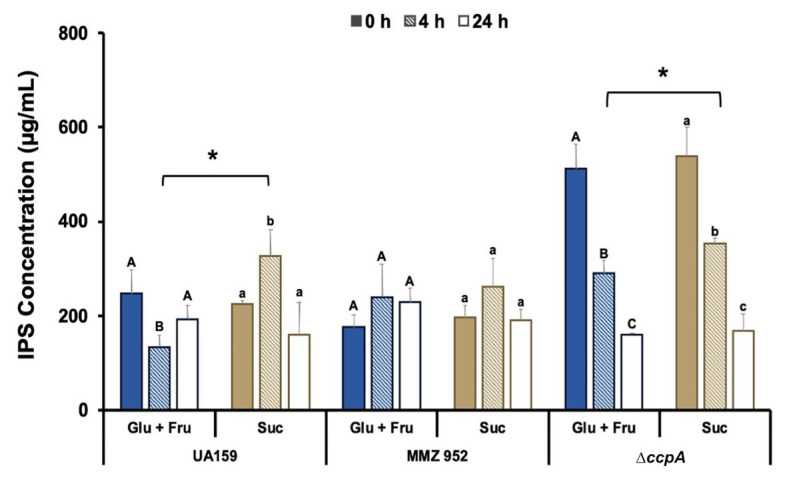
Measurements of IPS concentrations. Experiments were conducted using *S. mutans* UA159, MMZ952, and ∆*ccpA* cells grown on Glu+Fru or Suc, with samples taken at baseline (0h), and after 4 and 24h of starvation. For each strain, distinct capital letters (Glu+Fru) and lower case letters (Suc) indicate statistically significant effects in the harvest time factor, and asterisks indicate significant difference between carbohydrates for the same strain at the same time point (Two-way ANOVA, Mean±SD; *n*=3; *p*<0.05).

To evaluate the effects of carbohydrate exposure followed by limitation on IPS synthesis and degradation, IPS content was quantified using an iodine-based assay. IPS levels in Glu+Fru-grown WT cells were notably reduced after 4h of starvation, and remained slightly lower than at baseline at the 24-h time point. However, WT cells grown with sucrose showed the opposite effect: IPS levels increased after 4h of starvation, only to decrease again when starvation extended to 24h (Figure 3). In strain MMZ952, which lacks sucrolytic exo-enzymes, no significant change in IPS content was seen throughout the 24-h period, regardless of the carbohydrates used to culture the cells. We posit that cell-adherent EPS, perhaps glucans, can serve as a source for IPS accumulation during the starvation conditions used in this study. Collectively, these results suggest that metabolic activities of sucrolytic exo-enzymes help to enhance IPS storage in *S. mutans*, and potentially regulate degradative activities as well. When *ccpA* was deleted, *S. mutans* significantly increased IPS synthesis regardless of the carbohydrate source, doubling its production at baseline. Upon exposure to starvation conditions, however, a decline in IPS levels was observed as the *ccpA* mutant progressed through the 24-h starvation period.

**Figure 4:**
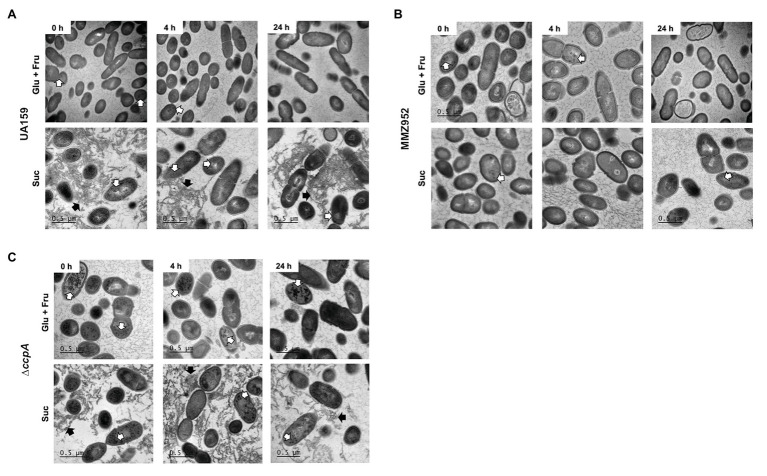
Transmission electron microscopy (TEM). Cells of *S. mutans* strains UA159 **(A)**, MMZ952 **(B)**, and ∆*ccpA* **(C)** grown with Glu+Fru or Suc were harvested at baseline (0h), and after 4 and 24h of carbohydrate starvation, followed by treatments to stain intracellular polysaccharides. The images show the presence of IPS (black dots, indicated by open arrows) in all groups, and extracellular polysaccharides (filled black arrows) in UA159 and ∆*ccpA* samples when grown with sucrose (Suc).

To support the IPS assay results, TEM was performed on strains cultured under the same conditions (Figure 4). The TEM images highlighted the enhanced ability of the CcpA-deficient mutant to accumulate IPS, regardless of growth carbohydrate. The heavily-stained dots of IPS were reduced after onset of starvation, in particular for the *∆ccpA* group, indicating IPS was degraded once external carbon sources were depleted. These results suggest that CcpA exerts a regulatory effect not only on the biosynthesis of IPS, but also on its degradation. Besides IPS, TEM images revealed the extracellular matrix and its EPS content in sucrose-grown cells, elements not found in Glu+Fru-grown cells and the MMZ952 group; adding support to the idea that cell-adherent EPS is the source of IPS under these conditions.

When bacterial viability was monitored during the same period, however, CFU enumeration revealed that the number of viable cells remained largely unchanged for each of these strains, regardless of the carbohydrates used for initial cultivation (Supplementary Figure S2). Additionally, no differences were found among strains in all time points evaluated. We posit that observation over longer periods of time is needed to discern the effects of IPS or EPS on viability.

### Measurements of Intracellular G1P and PTS Activities

**Figure 5:**
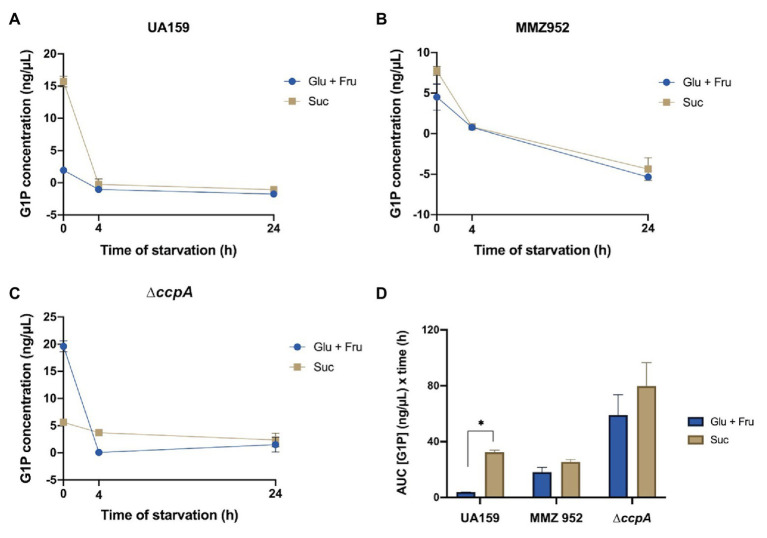
Measurements of G1P concentrations. Cell lysates of *S. mutans* strains UA159 **(A)**, MMZ952 **(B)**, and ∆*ccpA* **(C)** cultivated with Glu+Fru or Suc were prepared using cells harvested at baseline or after 4 or 24h of starvation. The G1P measurements over time (mean and SD as error bars; *n*=3) are presented as linked scatter plots **(A–C)**, and their area under the curve data calculated **(D)**. Asterisks indicate significant difference between carbohydrates (One-way ANOVA; *p*<0.05; Mean±SD; *n*=3).

To further the understanding of how cells respond to carbohydrates and starvation conditions, the levels of G1P, which is both a precursor required for IPS synthesis and a degradative product, were assessed as an indicator of glycogen metabolism (Figure 5). The amounts of G1P in UA159 and MMZ952 at baseline, but especially UA159, were markedly higher after growth in sucrose than in the control group (Glu+Fru). In contrast, the *ccpA*-deficient mutant grown with Glu+Fru produced the highest level of G1P among all strains at the baseline, but much lower G1P levels were detected in the same cells when grown with Suc. When exposed to starvation conditions, WT and MMZ952 had significantly reduced G1P concentrations, regardless of carbohydrate source, and the levels remained low at the 24-h time point. As an exception, relatively steady G1P concentrations were observed in sucrose-grown ∆*ccpA* cells after onset of carbohydrate starvation. When AUC was calculated (Figure 5D), the results indicated that neither MMZ952 nor ∆*ccpA* showed a significant difference in G1P levels between the two carbohydrate conditions over the course of the experiment (*p*>0.05).

**Figure 6:**
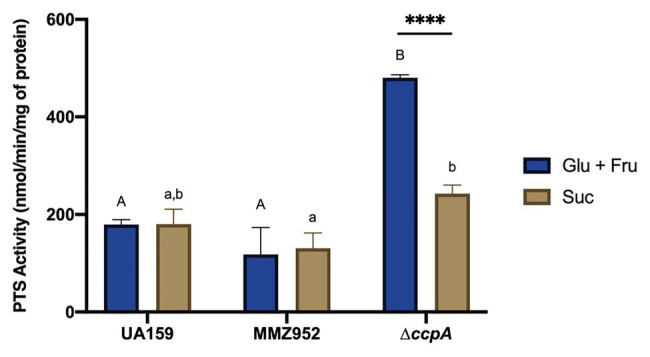
PEP-dependent sugar-specific PTS activities. Bacterial strains UA159, MMZ952, and ∆*ccpA* were grown in TY supplemented with Glu+Fru or Suc, followed by PTS assays measuring the transport of the same carbohydrates in which the cells were grown. Asterisks indicate significant difference in activities between carbohydrates for ∆*ccpA* (^****^ *p*<0.0001). Distinct letters indicate significant difference among the strains grown with Glu+Fru (capital) or Suc (lower case; Two-way ANOVA; *n*=3; *p*<0.05).

Phosphotransferase system assays were performed in order to see if some of these phenotypes observed so far were due to differences in the ability of these strains to internalize carbohydrates. For each experiment, the same carbohydrate was used in the PTS assay as what was used for cultivating the bacterium. The results revealed that CcpA-deficient strains, but not UA159 or MMZ952, had significantly higher PTS activity in the presence of Glu+Fru than Suc, when cultured using respective carbohydrates in the growth media (Figure 6; *p*<0.05). Interestingly, when Suc was used, PTS-activity in *∆ccpA* and UA159 did not differ (*p*>0.05). These results suggest CcpA may specifically regulate the glucose-PTS and fructose-PTS activities, consistent with our previous work indicating direct regulation of genes encoding both glucose- and fructose-PTS permeases (Zeng et al., 2017).

### Sucrolytic Exo-Enzymes and EPS Contribute to Acid Production in the Absence of Carbohydrates

**Figure 7:**
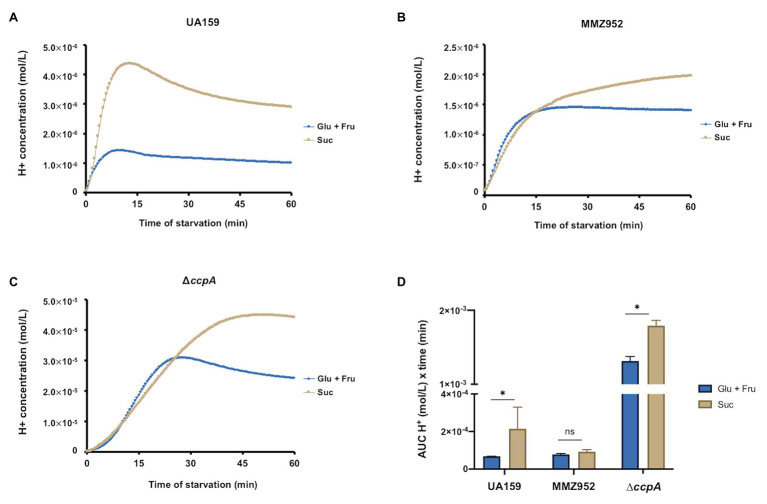
pH drop assay. H^+^ concentrations were converted from pH values measured using cell suspensions of *S. mutans* strains UA159 **(A)**, MMZ952 **(B)**, and ∆*ccpA* **(C)** cultured with Glu+Fru or Suc, and plotted against incubation time (*n*=2). Area under the curve was calculated for each dataset and presented in **(D)**. Asterisks indicate significant difference between carbohydrates (One-way ANOVA; *p*<0.05; Mean±SD; *n*=2).

The ability of *S. mutans* to release organic acids by fermenting carbohydrates is directly related to its cariogenic potential. To evaluate the capacity of *S. mutans* and its isogenic mutants to lower the pH by utilizing IPS or other storage carbohydrates, the pH of the cell suspension was monitored in the absence of added sugars. After 60min of incubation (Supplementary Figure S3), the WT UA159 cells prepared with sucrose reduced the environmental pH from near neutral (≈7.2) to approximately 5.5, which was significantly lower than the same cells grown on Glu+Fru (≈6.0). On the other hand, MMZ952 cells grown in Glu+Fru or Suc dropped the pH to similar levels (5.8–5.9; Supplementary Figure S3). Consistent with a previous study (Abranches et al., 2008), CcpA-deficient cells showed the greatest capacity to reduce the pH in the absence of exogenous carbohydrates, dropping the pH to as low as 4.3 when grown on Suc and to 4.7 when grown on Glu+Fru (Supplementary Figure S3). When these pH values were converted into proton (H^+^) concentrations to facilitate statistical analysis (Figure 7), it was clear that sucrose-grown cells generally released more H^+^ over time. AUC data analysis revealed such effects were dependent on the integrity of the sucrolytic exo-enzymes (Figure 7D). Further, since UA159 was shown at baseline to harbor similar levels of IPS when cultured under either sugar condition (Figure 3), the fact that the strain showed enhanced acidification of the cell suspensions during the pH drop on Suc suggests that, aside from IPS, other sources of energy storage, e.g., EPS, contributed to medium acidification. A similar behavior was seen with the *ccpA* mutant, but not MMZ952.

## Discussion

Glycogen-like IPS are important attributes for the persistence and virulence of *S. mutans*, particularly during fasting periods. Importantly, these energy storage compounds contribute to the initiation and progression of dental caries, as IPS synthesis allows the cells to capture and store a larger proportion of dietary carbohydrate, thereby extending the depth and duration of exposure of the tooth to a demineralizing environment (Harris et al., 1992; Busuioc et al., 2009). IPS can be formed from dietary carbohydrates, including sucrose. In this regard, we hypothesized that, besides the well-established roles of sucrose in enhancement of biofilm formation and virulence, acid production, and EPS synthesis (Rölla, 1989; Leme et al., 2006; Xiao and Koo, 2010; Xiao et al., 2012; Koo et al., 2013; Cai et al., 2018), that not-yet-explored sucrose dissimilation pathways could influence cariogenicity *via* the IPS system. Herein, it was discovered that the sucrolytic exo-enzymes of *S. mutans* had profound effects on physiology and gene regulation. Specifically, we showed that metabolism of sucrose *via* extracellular sucrolytic enzymes alters the expression of the *glg* operon in a manner that modulates IPS accumulation and the way in which cells metabolize IPS when subsequently faced with carbohydrate starvation. The changes in gene expression, and likely on the activity of some enzymes involved in carbohydrate utilization, induced by the EPS synthesis enzymes’ attack on sucrose manifest in enhanced IPS storage, increased intracellular G1P pools, and increased acid production in the absence of added sucrose. Interestingly, all these sucrose-dependent effects were accompanied by a delayed/subdued activation of the *glg* operon that encodes all IPS-metabolizing enzymes; anabolic and catabolic. These outcomes will likely enable *S. mutans* to persist better in dental biofilms, while allowing the continued catabolism of intracellular reserves to support growth and acid production even after depletion of exogenously supplied carbohydrates. Additionally, a primary regulator of central carbon metabolism and a regulator of *glg* gene expression, CcpA, was found to regulate IPS metabolism by reducing overall energy input into, and output from, IPS production; perhaps in favor of balancing the needs of other cellular functions that are required for persistence and virulence expression.

When presented with sucrose, *S. mutans* UA159 can uptake the disaccharide directly *via* a dedicated PTS permease (ScrA) and the trehalose PTS permease (Poy and Jacobson, 1990), phosphorylating and subsequently hydrolyzing the internalized sucrose-6-phosphate into glucose-6-phosphate (G6P) and fructose (Chassy and Porter, 1979; Slee and Tanzer, 1982; Tao et al., 1993). On the other hand, sucrose can be metabolized extracellularly by a fructosyltransferase and three glucosyltransferases that hydrolyze the bond between glucose and fructose, generating fructose-based and glucose-based homopolymers of various linkages, respectively, and releasing the other monosaccharide, which can then be reinternalized by the PTS (Colby and Russell, 1997; Bowen and Koo, 2011). Most *S. mutans* strains also produce a secreted β-fructosidase that can cleave sucrose into fructose and glucose, but the enzyme is usually produced at low levels unless cells are grown on fructose polymers. Other research on sucrose metabolism by oral biofilms also supports the potential creation of additional glucosides – e.g., the α1,3-linked disaccharide of glucose, nigerose – that can be utilized by *S. mutans* (Ajdic and Chen, 2013). In effect, when sucrose is provided as the sole carbohydrate source, it can yield an array of distinct species of carbohydrates available to *S. mutans*, namely sucrose, glucose, fructose, fructans, and soluble and insoluble glucans of varying molecular weights. Studies (Zeng and Burne, 2013, 2016) have illustrated the distinct impact of sucrose and fructose on *S. mutans*’ gene expression at the transcriptomic level. In this context, the effects of sucrose on IPS-related gene regulation and metabolism are the results of multiple types of carbohydrates being sensed and metabolized by *S. mutans*. Also of note, cells growing on sucrose do tend to aggregate, so we cannot exclude the possibility that intercellular contact or communication, or different microenvironments created by cell aggregation, did not influence the observations, we have made here. Still, utilizing a mutant derivative of UA159, MMZ952 that was engineered by mutating/deleting all known extracellular enzymes capable of targeting sucrose, thereby restricting sucrose utilization to PTS-dependent pathways, we showed that these extracellular enzymatic activities were likely responsible for significant IPS phenotypes directly related to virulence. At first glance, it may seem reasonable to suggest that restricting sucrose metabolism to intracellular routes should enhance the role of IPS in prolonging persistence and acid production, since the lack of EPS synthesis should make more sugar available for immediate internalization, catabolism, and IPS accumulation. Instead, growth of the MMZ952 on Glu+Fru or on Suc resulted in a temporary surge (at hour 4 post starvation) in *glg* transcription, but without the aforementioned benefits in accumulation of IPS and acid production. This negative effect on *glg* expression at the onset of starvation associated with exo-sucrase-dependent activities may reflect a strategy in carbon metabolism to achieve optimal survival, perhaps by reducing overflow metabolism. Since deletion of CcpA resulted in phenotypes dissimilar, and sometimes opposite, to that of MMZ952, it is clear that additional sucrose-dependent factor(s) are involved in regulation of IPS metabolism.

Synthesis of IPS in bacteria is a multi-reaction process that begins with the conversion of excess G6P into G1P by phosphoglucomutase (PGM; Figure 1). *Streptococcus mutans* maintains at least two orthologs of PGM-encoding genes, SMU.1077 and SMU.1747c, and both gene products, α-PGM and β-PGM, respectively, are potentially involved in this reaction (Buckley et al., 2014). G1P is then converted by GlgCD (ADP-Glc-PP) into ADP-glucose (Ballicora et al., 2003; Diez et al., 2013) that serves as the sugar-nucleotide donor for GlgA to elongate α-1,4 glucan chains (Preiss, 2009; Wilson et al., 2010). Formation of ADP-glucose is allosterically activated by high-energy metabolites, such as PEP, F6P, G6P, and F-1,6-bP, while the reaction itself is easily reversible and mostly driven by availability of the substrates (Preiss, 2006; Wilson et al., 2010). Degradation of IPS is carried out by a glycogen phosphorylase enzyme (*phsG/glgP*) that removes individual glucose moieties from the non-reducing end of the polysaccharides and releases G1P for glycolysis (Alonso-Casajús et al., 2006; Wilson et al., 2010; Sato et al., 2013). In accordance with this multi-step process, our study here suggested that regulation of IPS metabolism in *S. mutans* likely occurs at multiple levels. First, the apparent operon structure (*glgBCDAP*) facilitates co-expression of the mRNAs for both biosynthetic and degradative enzymes. Previous transcriptomic studies (Abranches et al., 2008; Zeng et al., 2013) and results from this report all indicated that the entire operon is likely regulated in the same direction, at least with regard to carbohydrate source or availability, or as a result of particular mutations in regulatory genes. Such genetic arrangement in theory would not allow differential regulation of anabolic and catabolic activities solely at the level of production of the mRNA. Thus, we consider differential regulation at post-transcription levels, e.g., mRNA stability or translation efficiency to be an important element governing IPS metabolism and the outcomes we observed here. Second, the temporal transcription profile of the *glg* operon under starvation conditions tends not to match the abundance of IPS accumulated by the bacterium. Transcription of *glg* genes in the WT in cells that were grown in Glu+Fru peaked shortly after the start of carbohydrate starvation, whereas the IPS levels showed a temporary drop at the same time point. Use of Suc in the WT culture reduced the early peak in mRNA levels; however, the highest IPS levels were present around the same time. The contrast in the *ccpA* mutant background was even more substantial. Nevertheless, the mRNA levels detected across all conditions at baseline appeared to be consistent with their respective IPS levels. Thus, it appears that during carbohydrate starvation, actual physiological parameters that are reflected in the levels of metabolic intermediates, such as G6P, G1P, ADP, and Pi, may play a more direct role in regulating IPS metabolism. In fact, it was previously observed that IPS formation does not occur simultaneously to the ADP-Glc-PP activity peak in *S. mutans* (Birkhed and Tanzer, 1979). Third are the impacts of CCR and IPS degradation. It may seem counterintuitive that an operon required for managing energy storage is induced under conditions of nutrient deprivation. Conversely, this observation may reflect that control of IPS metabolism is primarily exerted in response to the bioenergetic state of the cell, such as a state where energy conservation is critical. We have detected much higher amounts of IPS in the *ccpA* mutant at baseline relative to other CcpA-proficient stains, although the mRNA levels of the *glg* genes were only slightly elevated in the mutant (Figures 2, 3). We believe this was partly due to higher PTS activities in the *ccpA* mutant, resulting in increased intracellular pools of high-energy intermediates such as G6P and F6P (as is G1P, Figure 5C), thereby enhancing accumulation of IPS. Furthermore, in contrast to relatively consistent levels of IPS being detected during the 24-h period in the CcpA-proficient backgrounds, the *ccpA* mutant showed a rapid decline over time in IPS levels, demonstrating a role for CcpA in influencing IPS degradation at the enzymatic level, almost certainly in an indirect way by CcpA-dependent regulation of a factor(s) that modulates IPS use. Finally, upstream and often disparately regulated from the *glg* operon is *pulA* (SMU.1541), which encodes for a putative pullulanase (glycosidase family 13) required for persistence under starvation conditions (Busuioc et al., 2009). PulA could have a significant role in modifying IPS structure, e.g., as an intracellular de-branching enzyme for glycogen. Most *S. mutans* genomes also harbor another paralog of *glgP*, SMU.1564, that is part of the maltose (*mal*) utilization gene cluster (SMU.1564–1566) responsible for modifying, internalizing, and degrading certain α-glucans (Sato et al., 2013, 2015; Hobbs et al., 2018). There remains the possibility, therefore, that enzymes not encoded in the *glg* operon can be differentially regulated to influence IPS synthesis and degradation.

Results from our pH drop assays suggested that EPS storage contributes to acid production under starvation conditions. *Streptococcus mutans* produce both glucans and fructans from sucrose. Degradation of these EPS compounds depends on the presence of dextranases and fructanase in *S. mutans*, the activities of which have been shown to contribute to virulence (Staat and Schachtele, 1974; Burne et al., 1996; Khalikova et al., 2005; Klahan et al., 2018). Although the cells tested in our pH drop assays were washed with water beforehand, we reason that some EPS, glucans in particular, can remain in a cell-associated form and that some fraction of water-insoluble glucans may still be present in the cell pellet following washing of cells. These results also allowed us to posit that EPS accumulated before the start of starvation could provide hexoses or other oligosaccharides that can be internalized and converted to IPS. This would partly explain the increased IPS levels detected after 4h of starvation in UA159 cells grown on sucrose, and the absence of this increase in cells lacking the sucrolytic exo-enzyme. Fructans, on the other hand, are generally considered more water-soluble than glucans and not bound by cell envelope-associated proteins, and therefore are likely to have been lost during washing of cells. As such, we are expecting a negligible contribution of fructans to IPS synthesis and related acidification as observed in this study, although a more significant role of fructans clearly exists *in vivo*. Furthermore, a microarray study on a glucose-PTS (*manLMNO*) mutant of UA159 has found derepressed expression of the *glg* operon (Abranches et al., 2006). Considering the free glucose being liberated by dextranase from glucans, there exists the possibility that transcription of the *glg* operon is regulated by both PTS-dependent and CcpA-dependent CCR, the former acting under low carbohydrate conditions and the latter higher (>5mM levels; Zeng and Burne, 2008). Because CcpA also negatively regulates the *manLMNO* operon, it has been reported that CcpA-independent CCR can be enhanced in the *ccpA* mutant due to elevated glucose-PTS activities (Zeng and Burne, 2008). The observation that Suc-grown *ccpA* mutant had higher *glg* expression at baseline (high carbohydrate levels), yet a lack of activation at 4h of starvation (low carbohydrate levels) matches the expression pattern of genes regulated in this manner (Zeng and Burne, 2008). This mode of regulation is also consistent with a previous study linking *glg* expression with PTS-dependent activities (Spatafora et al., 1999). Further research is needed to test this hypothesis.

In conclusion, our study revealed that as sucrose is metabolized by the EPS synthetic machinery of *S. mutans* UA159, it can have a profound influence on IPS metabolism during starvation. EPS has long been understood to be a major factor contributing to the virulence of *S. mutans* and this work reveals a potentially new mechanism for enhancement of virulence: EPS synthetic enzymes influence IPS metabolism by enhancing IPS storage and prolonging acid production during starvation. Future research should focus on identifying molecular mechanisms directly responsible for these effects, and doing so in a more complex biofilm setting that involves other abundant constituents of the dental microbiome.

## Data Availability Statement

The original contributions presented in the study are included in the article/Supplementary Material; further inquiries can be directed to the corresponding author.

## Author Contributions

RB, BC, and LZ: conception and experiment design. RB and LZ: methodology. BC: experiment performance. BC, AR, and LZ: data analysis. RB: materials and software. RB, LZ, and AR: funding acquisition. BC and LZ: manuscript drafting. LZ, RB, and AR: review and editing. All authors contributed to the article and approved the submitted version.

### Conflict of Interest

The authors declare that the research was conducted in the absence of any commercial or financial relationships that could be construed as a potential conflict of interest.

## References

[ref1] Abranches J. Candella M. M. Wen Z. T. Baker H. V. Burne R. A. (2006). Different roles of EIIAB^Man^ and EII^Glc^ in regulation of energy metabolism, biofilm development, and competence in *Streptococcus mutans*. J. Bacteriol. 188, 3748–3756. 10.1128/JB.00169-06, PMID: 16707667 PMC1482907

[ref2] Abranches J. Nascimento M. M. Zeng L. Browngardt C. M. Wen Z. T. Rivera M. F.. (2008). CcpA regulates central metabolism and virulence gene expression in *Streptococcus mutans*. J. Bacteriol. 190, 2340–2349. 10.1128/JB.01237-07, PMID: 18223086 PMC2293215

[ref3] Ahn S. J. Ahn S. J. Browngardt C. M. Burne R. A. (2009). Changes in biochemical and phenotypic properties of *Streptococcus mutans* during growth with aeration. Appl. Environ. Microbiol. 75, 2517–2527. 10.1128/AEM.02367-08, PMID: 19251884 PMC2675223

[ref4] Ahn S. J. Lemos J. A. Burne R. A. (2005). Role of HtrA in growth and competence of *Streptococcus mutans* UA159. J. Bacteriol. 187, 3028–3038. 10.1128/JB.187.9.3028-3038.2005, PMID: 15838029 PMC1082816

[ref5] Ajdic D. Chen Z. (2013). A novel phosphotransferase system of *Streptococcus mutans* is responsible for transport of carbohydrates with alpha-1,3 linkage. Mol. Oral Microbiol. 28, 114–128. 10.1111/omi.12009, PMID: 23193985 PMC3593818

[ref6] Alonso-Casajús N. Dauvillée D. Viale A. M. Muñoz F. J. Baroja-Fernández E. Morán-Zorzano M. T.. (2006). Glycogen phosphorylase, the product of the *glgP* gene, catalyzes glycogen breakdown by removing glucose units from the nonreducing ends in *Escherichia coli*. J. Bacteriol. 188, 5266–5272. 10.1128/JB.01566-05, PMID: 16816199 PMC1539952

[ref7] Ballicora M. A. Iglesias A. A. Preiss J. (2003). ADP-glucose pyrophosphorylase, a regulatory enzyme for bacterial glycogen synthesis. Microbiol. Mol. Biol. Rev. 67, 213–225. 10.1128/mmbr.67.2.213-225.2003, PMID: 12794190 PMC156471

[ref8] Bender G. R. Sutton S. V. Marquis R. E. (1986). Acid tolerance, proton permeabilities, and membrane ATPases of oral streptococci. Infect. Immun. 53, 331–338. 10.1128/IAI.53.2.331-338.1986, PMID: 3015800 PMC260879

[ref9] Birkhed D. Tanzer J. M. (1979). Glycogen synthesis pathway in *Streptococcus mutans* strain NCTC 10449S and its glycogen synthesis-defective mutant 805. Arch. Oral Biol. 24, 67–73. 10.1016/0003-9969(79)90177-8, PMID: 116633

[ref10] Bowen W. H. Koo H. (2011). Biology of *Streptococcus mutans*-derived glucosyltransferases: role in extracellular matrix formation of cariogenic biofilms. Caries Res. 45, 69–86. 10.1159/000324598, PMID: 21346355 PMC3068567

[ref11] Buckley A. A. Faustoferri R. C. Quivey R. G. (2014). β-phosphoglucomutase contributes to aciduricity in *Streptococcus mutans*. Microbiology 160, 818–827. 10.1099/mic.0.075754-0, PMID: 24509501 PMC3973451

[ref12] Burne R. A. Chen Y. Y. Wexler D. L. Kuramitsu H. Bowen W. H. (1996). Cariogenicity of *Streptococcus mutans* strains with defects in fructan metabolism assessed in a program-fed specific-pathogen-free rat model. J. Dent. Res. 75, 1572–1577. 10.1177/00220345960750080801, PMID: 8906125

[ref13] Busuioc M. Mackiewicz K. Buttaro B. A. Piggot P. J. (2009). Role of intracellular polysaccharide in persistence of *Streptococcus mutans*. J. Bacteriol. 191, 7315–7322. 10.1128/jb.00425-09, PMID: 19801415 PMC2786568

[ref14] Cai J. N. Jung J. E. Lee M. H. Choi H. M. Jeon J. G. (2018). Sucrose challenges to *Streptococcus mutans* biofilms and the curve fitting for the biofilm changes. FEMS Microbiol. Ecol. 94. 10.1093/femsec/fiy091, PMID: 29788432

[ref15] Carlsson J. (1983). “Regulation of sugar metabolism in relation to feast-and-famine existence of plaque” in Cariology today. ed. Guggenheim B. (Basel: Karger), 205–211.

[ref16] Chassy B. M. Porter E. V. (1979). Initial characterization of sucrose-6-phosphate hydrolase from *Streptococcus mutans* and its apparent identity with intracellular invertase. Biochem. Biophys. Res. Commun. 89, 307–314. 10.1016/0006-291x(79)90979-3, PMID: 224874

[ref17] Colby S. M. Russell R. R. B. (1997). Sugar metabolism by mutans streptococci. J. Appl. Microbiol. 83, 80S–88S. 10.1046/j.1365-2672.83.s1.9.x, PMID: 28621891

[ref18] Demonte A. M. Diez M. D. A. Naleway C. Iglesias A. A. Ballicora M. A. (2017). Monofluorophosphate blocks internal polysaccharide synthesis in *Streptococcus mutans*. PLoS One 12:e0170483. 10.1371/journal.pone.0170483, PMID: 28125652 PMC5268466

[ref19] Diez M. D. A. Demonte A. M. Guerrero S. A. Ballicora M. A. Iglesias A. A. (2013). The ADP-glucose pyrophosphorylase from *Streptococcus mutans* provides evidence for the regulation of polysaccharide biosynthesis in firmicutes. Mol. Microbiol. 90, 1011–1027. 10.1111/mmi.12413, PMID: 24112771

[ref20] DiPersio J. R. Mattingly S. J. Higgins M. L. Shockman G. D. (1974). Measurement of intracellular iodophilic polysaccharide in two cariogenic strains of *Streptococcus mutans* by cytochemical and chemical methods. Infect. Immun. 10, 597–604. 10.1128/IAI.10.3.597-604.1974, PMID: 4139118 PMC422994

[ref21] Gibbons R. J. Socransky S. S. (1962). Intracellular polysaccharide storage by organisms in dental plaques. Its relation to dental caries and microbial ecology of the oral cavity. Arch. Oral Biol. 7, 73–79. 10.1016/0003-9969(62)90050-X, PMID: 13898350

[ref22] Harris G. S. Michalek S. M. Curtiss R. 3rd. (1992). Cloning of a locus involved in *Streptococcus mutans* intracellular polysaccharide accumulation and virulence testing of an intracellular polysaccharide-deficient mutant. Infect. Immun. 60, 3175–3185. 10.1128/IAI.60.8.3175-3185.1992, PMID: 1322367 PMC257299

[ref23] Hobbs J. K. Pluvinage B. Boraston A. B. (2018). Glycan-metabolizing enzymes in microbe-host interactions: the *Streptococcus pneumoniae* paradigm. FEBS Lett. 592, 3865–3897. 10.1002/1873-3468.13045, PMID: 29608212

[ref24] Huis In’t Veld J. H. Backer Dirks O. (1978). Intracellular polysaccharide metabolism in *Streptococcus mutans*. Caries Res. 12, 243–249. 10.1159/000260340, PMID: 279404

[ref25] Khalikova E. Susi P. Korpela T. (2005). Microbial dextran-hydrolyzing enzymes: fundamentals and applications. Microbiol. Mol. Biol. Rev. 69, 306–325. 10.1128/MMBR.69.2.306-325.2005, PMID: 15944458 PMC1197420

[ref26] Kim E. J. Ryu S. I. Bae H. A. Huong N. T. Lee S. B. (2008). Biochemical characterisation of a glycogen branching enzyme from *Streptococcus mutans*: enzymatic modification of starch. Food Chem. 110, 979–984. 10.1016/j.foodchem.2008.03.025, PMID: 26047289

[ref27] Klahan P. Okuyama M. Jinnai K. Ma M. Kikuchi A. Kumagai Y.. (2018). Engineered dextranase from *Streptococcus mutans* enhances the production of longer isomaltooligosaccharides. Biosci. Biotechnol. Biochem. 82, 1480–1487. 10.1080/09168451.2018.1473026, PMID: 29806555

[ref28] Koo H. Falsetta M. L. Klein M. I. (2013). The exopolysaccharide matrix: a virulence determinant of cariogenic biofilm. J. Dent. Res. 92, 1065–1073. 10.1177/0022034513504218, PMID: 24045647 PMC3834652

[ref29] LeBlanc D. J. Crow V. L. Lee L. N. Garon C. F. (1979). Influence of the lactose plasmid on the metabolism of galactose by *Streptococcus lactis*. J. Bacteriol. 137, 878–884. 10.1128/JB.137.2.878-884.1979, PMID: 106044 PMC218370

[ref30] Leme A. F. P. Koo H. Bellato C. M. Bedi G. Cury J. A. (2006). The role of sucrose in cariogenic dental biofilm formation--new insight. J. Dent. Res. 85, 878–887. 10.1177/154405910608501002, PMID: 16998125 PMC2257872

[ref31] Moye Z. D. Zeng L. Burne R. A. (2014). Modification of gene expression and virulence traits in *Streptococcus mutans* in response to carbohydrate availability. Appl. Environ. Microbiol. 80, 972–985. 10.1128/aem.03579-13, PMID: 24271168 PMC3911228

[ref32] Oliveira B. E. C. Cury J. A. Ricomini Filho A. P. (2017). Biofilm extracellular polysaccharides degradation during starvation and enamel demineralization. PLoS One 12:e0181168. 10.1371/journal.pone.0181168, PMID: 28715508 PMC5513492

[ref33] Poy F. Jacobson G. R. (1990). Evidence that a low-affinity sucrose phosphotransferase activity in *Streptococcus mutans* GS-5 is a high-affinity trehalose uptake system. Infect. Immun. 58, 1479–1480. 10.1128/IAI.58.5.1479-1480.1990, PMID: 2323827 PMC258652

[ref34] Preiss J. (2006). “Bacterial glycogen inclusions: enzymology and regulation of synthesis” in Inclusions in prokaryotes. ed. Shiverly J. M. (Berlin: Spinger), 71–108.

[ref35] Preiss J. (2009). Glycogen: biosynthesis and regulation. EcoSal Plus 3. 10.1128/ecosalplus.4.7.4, PMID: 26443753

[ref500] Rocha D. J. Santos C. S. Pacheco L. G. (2015). Bacterial reference genes for gene expression studies by RT-qPCR: survey and analysis. Antonie van Leeuwenhoek. 108, 685–693. 10.1007/s10482-015-0524-1, PMID: 26149127

[ref36] Rölla G. (1989). Why is sucrose so cariogenic? The role of glucosyltransferase and polysaccharides. Scand. J. Oral Sci. 97, 115–119. 10.1111/j.1600-0722.1989.tb01439.x, PMID: 2523085

[ref37] Sampaio A. A. Souza S. E. Ricomini-Filho A. P. Cury A. A. D. B. Cavalcanti Y. W. Cury J. A. (2019). *Candida albicans* increases dentine demineralization provoked by *Streptococcus mutans* biofilm. Caries Res. 53, 322–331. 10.1159/000494033, PMID: 30448846

[ref38] Sato Y. Okamoto-Shibayama K. Azuma T. (2013). The *malQ* gene is essential for starch metabolism in *Streptococcus mutans*. J. Oral Microbiol. 521285. 10.3402/jom.v5i0.21285, PMID: 23930155 PMC3737437

[ref39] Sato Y. Okamoto-Shibayama K. Azuma T. (2015). Glucose-PTS involvement in maltose metabolism by *Streptococcus mutans*. Bull. Tokyo Dent. Coll. 56, 93–103. 10.2209/tdcpublication.56.93, PMID: 26084997

[ref40] Schmittgen T. D. Livak K. J. (2008). Analyzing real-time PCR data by the comparative C_T_ method. Nat. Protoc. 3, 1101–1108. 10.1038/nprot.2008.73, PMID: 18546601

[ref41] Slee A. M. Tanzer J. M. (1982). Sucrose transport by *Streptococcus mutans*. Evidence for multiple transport systems. Biochim. Biophys. Acta 692, 415–424. 10.1016/0005-2736(82)90392-3, PMID: 7171603

[ref42] Spatafora G. Rohrer K. Barnard D. Michalek S. (1995). A *Streptococcus mutans* mutant that synthesizes elevated levels of intracellular polysaccharide is hypercariogenic in vivo. Infect. Immun. 63, 2556–2563. 10.1128/IAI.63.7.2556-2563.1995, PMID: 7790069 PMC173342

[ref43] Spatafora G. A. Sheets M. June R. Luyimbazi D. Howard K. Hulbert R.. (1999). Regulated expression of the *Streptococcus mutans dlt* genes correlates with intracellular polysaccharide accumulation. J. Bacteriol. 181, 2363–2372. 10.1128/JB.181.8.2363-2372.1999, PMID: 10197997 PMC93659

[ref44] Staat R. H. Schachtele C. F. (1974). Evaluation of dextranase production by the cariogenic bacterium *Streptococcus mutans*. Infect. Immun. 9, 467–469. 10.1128/IAI.9.2.467-469.1974, PMID: 4816468 PMC414825

[ref45] Tao L. Sutcliffe I. C. Russell R. R. Ferretti J. J. (1993). Transport of sugars, including sucrose, by the *msm* transport system of *Streptococcus mutans*. J. Dent. Res. 72, 1386–1390. 10.1177/00220345930720100701, PMID: 8408880

[ref600] Vujanac M. Iyer V. S. Sengupta M. Ajdic D. (2015). Regulation of Streptococcus mutans PTSBio by the transcriptional repressor NigR. Mol. Oral Microbiol. 30, 280–294. 10.1111/omi.12093, PMID: 25580872 PMC4491040

[ref46] Wen Z. T. Burne R. A. (2002). Analysis of *cis*- and *trans*-acting factors involved in regulation of the *Streptococcus mutans* fructanase gene (*fruA*). J. Bacteriol. 184, 126–133. 10.1128/jb.184.1.126-133.2002, PMID: 11741852 PMC134753

[ref47] Wilson W. A. Roach P. J. Montero M. Baroja-Fernandez E. Munoz F. J. Eydallin G.. (2010). Regulation of glycogen metabolism in yeast and bacteria. FEMS Microbiol. Rev. 34, 952–985. 10.1111/j.1574-6976.2010.00220.x, PMID: 20412306 PMC2927715

[ref48] Xiao J. Klein M. I. Falsetta M. L. Lu B. Delahunty C. M. Yates J. R. 3rd.. (2012). The exopolysaccharide matrix modulates the interaction between 3D architecture and virulence of a mixed-species oral biofilm. PLoS Pathog. 8:e1002623. 10.1371/journal.ppat.1002623, PMID: 22496649 PMC3320608

[ref49] Xiao J. Koo H. (2010). Structural organization and dynamics of exopolysaccharide matrix and microcolonies formation by *Streptococcus mutans* in biofilms. J. Appl. Microbiol. 108, 2103–2113. 10.1111/j.1365-2672.2009.04616.x, PMID: 19941630

[ref50] Zeng L. Burne R. A. (2008). Multiple sugar: phosphotransferase system permeases participate in catabolite modification of gene expression in *Streptococcus mutans*. Mol. Microbiol. 70, 197–208. 10.1111/j.1365-2958.2008.06403.x, PMID: 18699864 PMC2583961

[ref51] Zeng L. Burne R. A. (2013). Comprehensive mutational analysis of sucrose-metabolizing pathways in *Streptococcus mutans* reveals novel roles for the sucrose phosphotransferase system permease. J. Bacteriol. 195, 833–843. 10.1128/JB.02042-12, PMID: 23222725 PMC3562097

[ref52] Zeng L. Burne R. A. (2016). Sucrose- and fructose-specific effects on the transcriptome of *Streptococcus mutans*, as determined by RNA sequencing. Appl. Environ. Microbiol. 82, 146–156. 10.1128/aem.02681-15, PMID: 26475108 PMC4702655

[ref53] Zeng L. Chakraborty B. Farivar T. Burne R. A. (2017). Coordinated regulation of the EII^Man^ and *fruRKI* operons of *Streptococcus mutans* by global and fructose-specific pathways. Appl. Environ. Microbiol. 83, e01403–e01417. 10.1128/AEM.01403-17, PMID: 28821551 PMC5648919

[ref54] Zeng L. Choi S. C. Danko C. G. Siepel A. Stanhope M. J. Burne R. A. (2013). Gene regulation by CcpA and catabolite repression explored by RNA-Seq in *Streptococcus mutans*. PLoS One 8:e60465. 10.1371/journal.pone.0060465, PMID: 23555977 PMC3610829

