# Supplementary material for: The Route of Sucrose Utilization by Streptococcus mutans Affects Intracellular Polysaccharide Metabolism
Source: Front Microbiol. 2021 Feb 2;12:636684. doi: 10.3389/fmicb.2021.636684 (PMC7884614; doi:10.3389/fmicb.2021.636684)
Supplement: Supplementary file 1 [file Data_Sheet_1.pdf]

Supplemental Materials to "The Route of Sucrose Utilization by *Streptococcus mutans* Affects Intracellular Polysaccharide Metabolism"

Bárbara Emanoele Costa Oliveira [1,2], Antônio Pedro Ricomini Filho[2], Robert A. Burne[1], Lin Zeng[1*].

[1] Department of Oral Biology, University of Florida, College of Dentistry, Gainesville, FL, USA.

[2] Department of Biosciences, Piracicaba Dental School, University of Campinas, Piracicaba, Brazil.

Includes Tables S1 to S2, and Figures S1 to S3.

Table S1. Relative message levels of the *glg* operon during carbohydrate starvation of three *S. mutans* strains, compared among different time points, for each carbohydrate condition (Two-way ANOVA; n = 3; p <0.05). ns, not significant.

Gene	Comparison	Glucose + Fructose		Sucrose	
		Mean Diff (Log2)	*P* Value	Mean Diff (Log2)	*P* Value
		S. mutans UA159			
glgA	0 vs. 4	-4.658	<0.0001	-0.9886	ns
	0 vs. 24	-1.358	ns	-3.098	0.0018
glgB	0 vs. 4	-4.120	<0.0001	-1.396	ns
	0 vs. 24	-1.051	ns	-2.277	0.0209
glgC	0 vs. 4	-13.05	<0.0001	-2.500	0.0111
	0 vs. 24	-4.544	<0.0001	-6.718	<0.0001
glgD	0 vs. 4	-7.012	<0.0001	-1.136	ns
	0 vs. 24	-1.986	0.0247	-3.482	0.0005
phsG	0 vs. 24	-3.183	0.0004	-0.6675	ns
	0 vs. 24	-1.540	ns	-2.467	0.0122
		MMZ952			
glgA	0 vs. 4	-5.416	<0.0001	-5.065	<0.0001
	0 vs. 24	-2.413	ns	-2.135	0.0347
glgB	0 vs. 4	-4.368	0.0005	-4.677	<0.0001
	0 vs. 24	-1.585	ns	-1.557	ns
glgC	0 vs. 4	-14.30	<0.0001	-13.34	<0.0001
	0 vs. 24	-4.910	0.0001	-4.446	<0.0001
glgD	0 vs. 4	-7.870	<0.0001	-7.247	<0.0001
	0 vs. 24	-2.766	0.0246	-2.573	0.0103
phsG	0 vs. 24	-3.874	0.0017	-3.331	0.0010
	0 vs. 24	-2.440	0.0496	-1.997	0.0495
		**Δ*ccpA***			
glgA	0 vs. 4	-6.648	<0.0001	-0.4379	ns
	0 vs. 24	-1.678	ns	-1.577	0.0005
glgB	0 vs. 4	-5.413	<0.0001	-0.0478	ns
	0 vs. 24	-1.294	ns	-1.520	0.0008
glgC	0 vs. 4	-14.01	<0.0001	-0.3756	ns
	0 vs. 24	-2.172	ns	-4.159	<0.0001
glgD	0 vs. 4	-7.959	<0.0001	-0.5249	ns
	0 vs. 24	-1.413	ns	-1.945	<0.0001
phsG	0 vs. 24	-4.103	0.0004	-0.3107	ns
	0 vs. 24	-0.4321	ns	-1.029	0.0214

Table S2. Relative message levels of the *glg* operon during carbohydrate starvation, compared among three *S. mutans* strains (UA = UA159, MMZ = MMZ952), under identical carbohydrate conditions (Two-way ANOVA; n = 3; p <0.05). ns, not significant.

Gene	***Comparison***	***Glucose + Fructose***		***Sucrose***	
		**Mean Diff (Log2)**	***P* Value**	**Mean Diff (Log2)**	***P* Value**
0 h of starvation					
glgA	UA vs. MMZ	0.6878	0.0248	0.05551	ns
	UA vs. ΔccpA	-0.1027	ns	-1.248	<0.0001
	MMZ vs. ΔccpA	-0.7905	0.0091	-1.304	<0.0001
glgB	UA vs. MMZ	0.5962	ns	-0.0217	ns
	UA vs. ΔccpA	-0.8763	0.0037	-1.560	<0.0001
	MMZ vs. ΔccpA	-1.473	<0.0001	-1.538	<0.0001
glgC	UA vs. MMZ	0.3112	ns	0.0896	ns
	UA vs. ΔccpA	-2.019	<0.0001	-2.766	<0.0001
	MMZ vs. ΔccpA	-2.330	<0.0001	-2.855	<0.0001
glgD	UA vs. MMZ	0.6950	0.0231	0.0413	ns
	UA vs. ΔccpA	-0.4750	ns	-1.571	<0.0001
	MMZ vs. ΔccpA	-1.170	0.0001	-1.612	<0.0001
phsG	UA vs. MMZ	0.7429	0.0146	0.0796	ns
	UA vs. ΔccpA	-0.1706	ns	-0.7932	0.0035
	MMZ vs. ΔccpA	-0.9135	0.0025	-0.8728	0.0013
4 h of starvation					
glgA	UA vs. MMZ	-0.0704	ns	-4.021	0.0002
	UA vs. ΔccpA	-2.093	ns	-0.6976	ns
	MMZ vs. ΔccpA	-2.023	ns	3.323	0.0019
glgB	UA vs. MMZ	0.3483	ns	-3.302	0.0020
	UA vs. ΔccpA	-2.169	ns	-0.2115	ns
	MMZ vs. ΔccpA	-2.518	ns	3.091	0.0038
glgC	UA vs. MMZ	-0.9392	ns	-10.75	<0.0001
	UA vs. ΔccpA	-2.989	ns	-0.6417	ns
	MMZ vs. ΔccpA	-2.050	ns	10.11	<0.0001
glgD	UA vs. MMZ	-0.1626	ns	-6.070	<0.0001
	UA vs. ΔccpA	-1.422	ns	-0.9595	ns
	MMZ vs. ΔccpA	-1.259	ns	5.110	<0.0001
phsG	UA vs. MMZ	0.0520	ns	-2.584	0.0162
	UA vs. ΔccpA	-1.090	ns	-0.4364	ns
	MMZ vs. ΔccpA	-1.142	ns	2.147	ns
24 h of starvation					
glgA	UA vs. MMZ	-0.3676	ns	1.018	ns
	UA vs. ΔccpA	-0.4227	ns	0.2721	ns
	MMZ vs. ΔccpA	-0.05515	ns	-0.7461	ns
glgB	UA vs. MMZ	0.06214	ns	0.6981	ns
	UA vs. ΔccpA	-1.119	ns	-0.8033	ns
	MMZ vs. ΔccpA	-1.181	ns	-1.501	ns
glgC	UA vs. MMZ	-0.0545	ns	2.362	0.0310
	UA vs. ΔccpA	0.3526	ns	-0.2068	ns
	MMZ vs. ΔccpA	0.4072	ns	-2.569	0.0179
glgD	UA vs. MMZ	-0.08514	ns	0.9501	ns
	UA vs. ΔccpA	0.09717	ns	-0.0332	ns
	MMZ vs. ΔccpA	0.1823	ns	-0.9833	ns
phsG	UA vs. MMZ	-0.1564	ns	0.5491	ns
	UA vs. ΔccpA	0.9376	ns	0.6439	ns
	MMZ vs. ΔccpA	1.094	ns	0.0948	ns

Fig S1. Growth curves of *S. mutans* strains UA159 (diamond), MMZ952 (square) and *ccpA::em* (triangle) grown on TY supplemented with 0.5% of glucose and 0.5% of fructose (A), or 1% of sucrose (B).

A

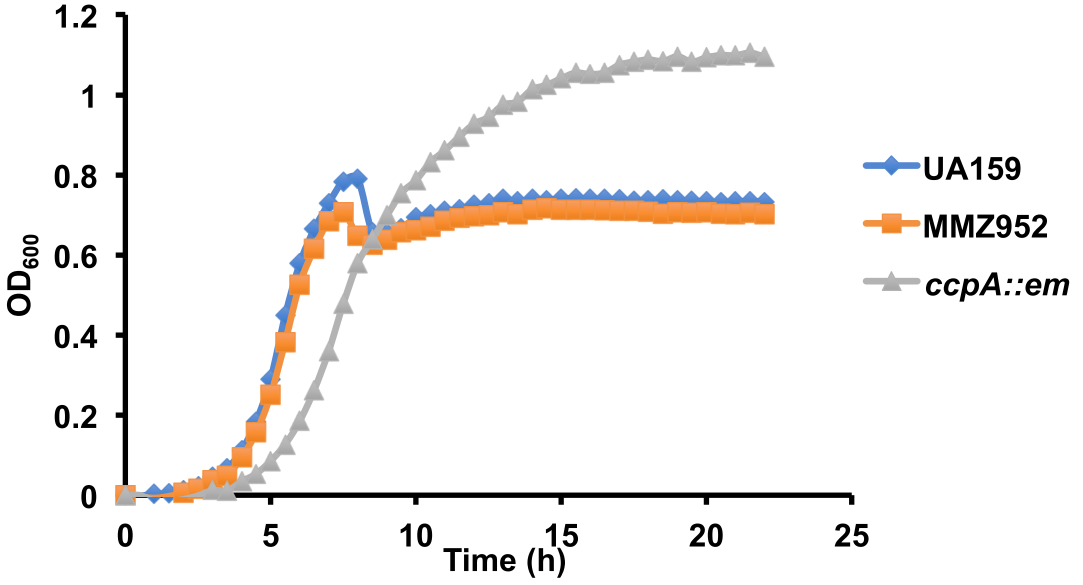

B

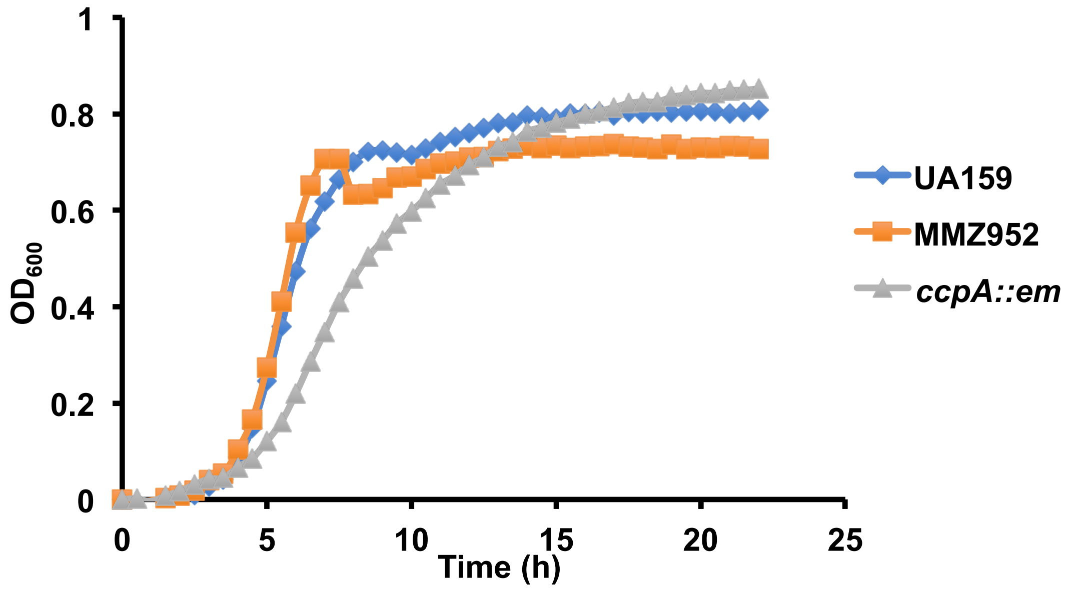

Fig S2. Enumeration of CFU in bacterial cultures of *S. mutans* strains UA159, MMZ952, and Δ*ccpA* harvested at baseline (0 h), and 4 h and 24 h after Glu + Fru (GF) or Suc interruption (Two-way ANOVA; Mean ± SD; n = 3; *p* >0.05).

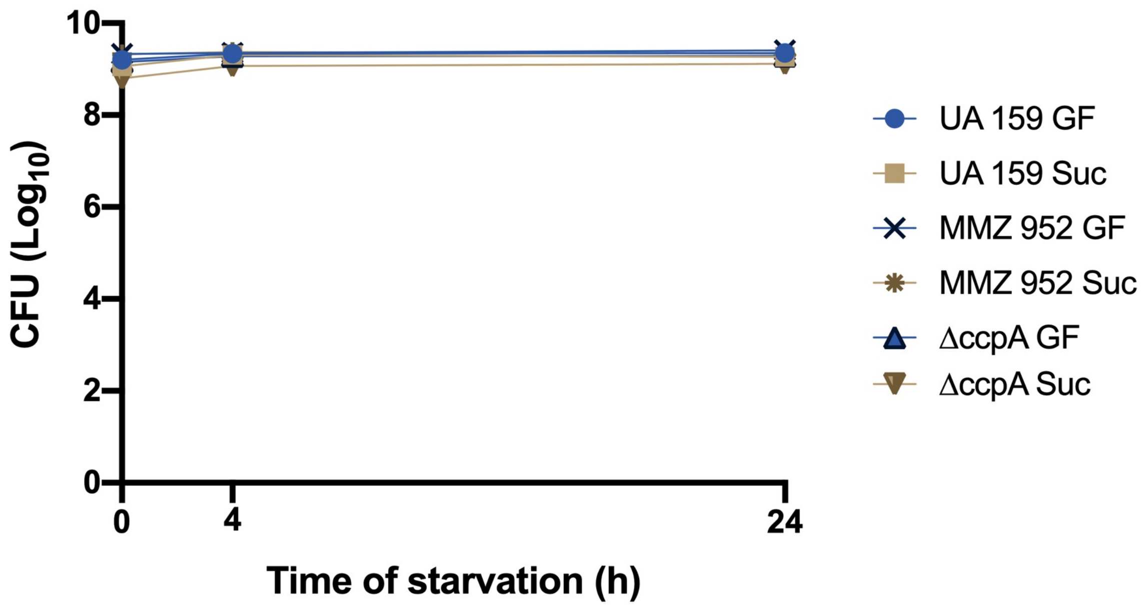

Fig S3. pH drop performed using cells of *S. mutans* strains UA159 (A), MMZ952 (B) and Δ*ccpA* (C) grown on Glu + Fru or Suc, in the absence of exogenous energy sources. Results shown are each the average of two independent experiments.

A

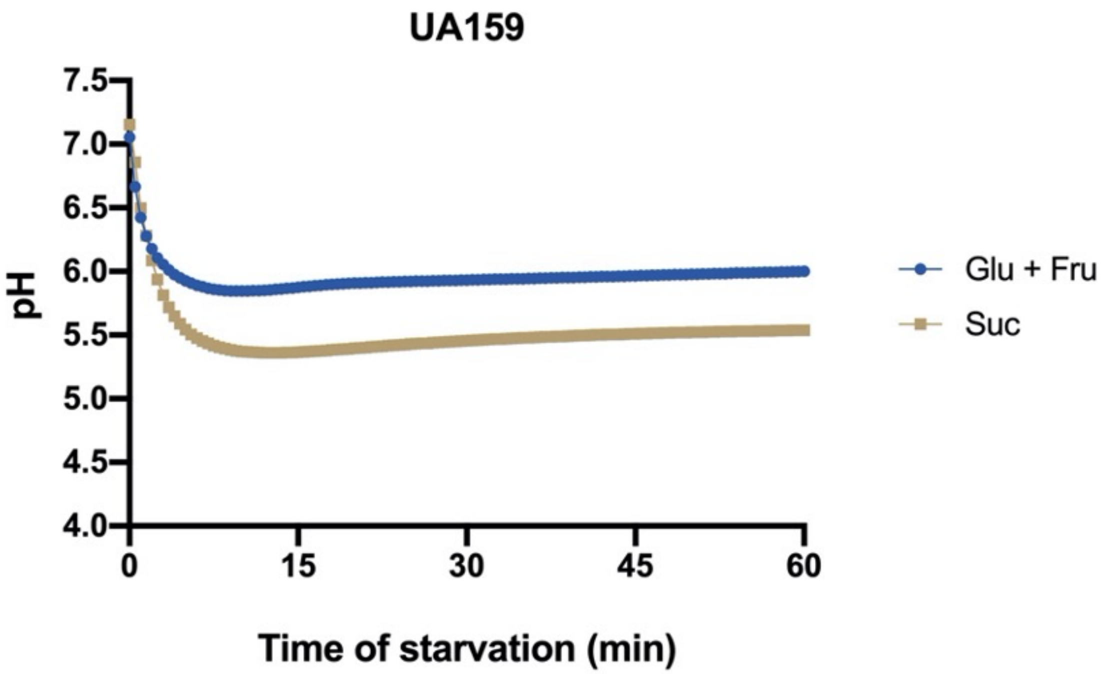

B

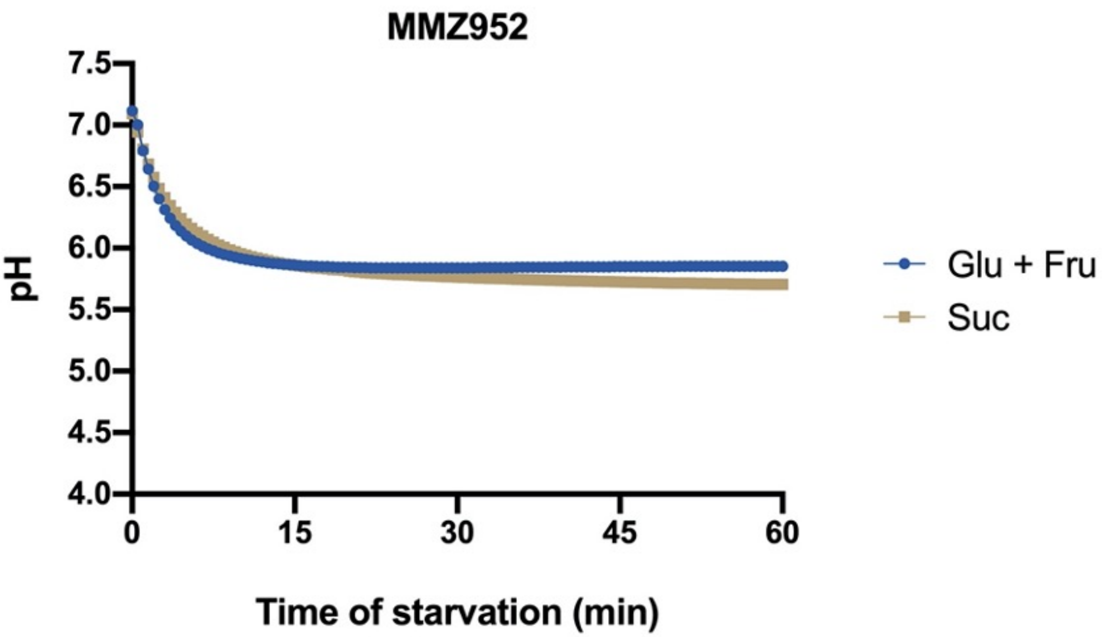

C

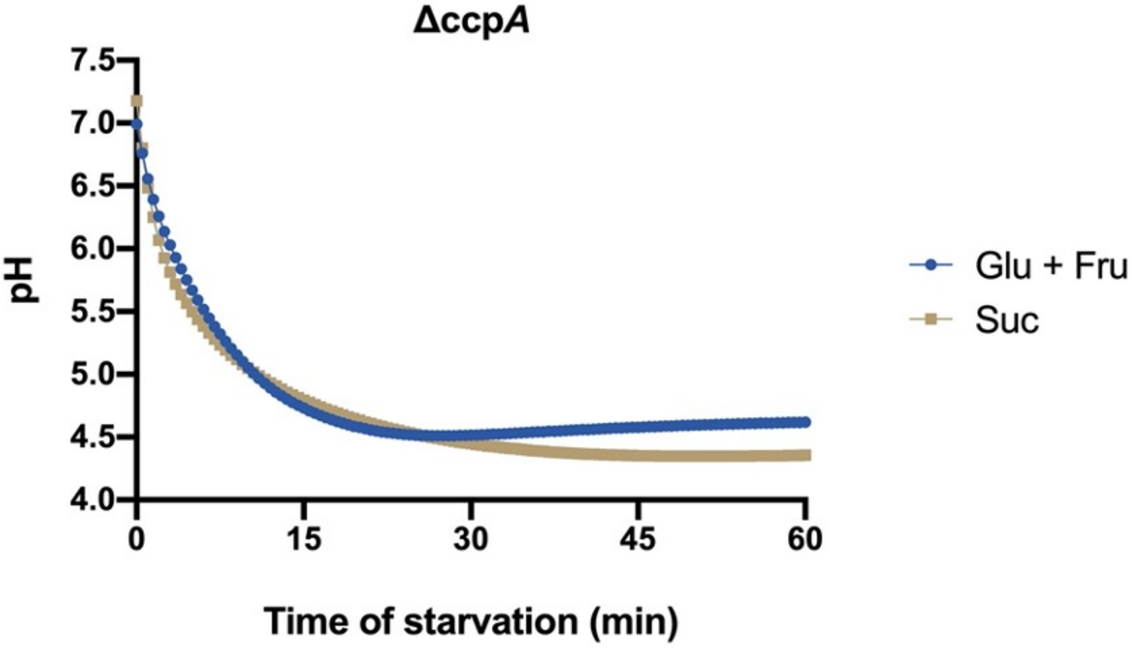
ΔccpA
7.5
7.0
6.5
6.0
5.5
5.0
4.5
4.0
pH
0
15
30
45
60
Time of starvation (min)
Glu + Fru
Suc
